# Indirect computed tomography lymphography identifies lymph node metastasis in rabbit pyriform sinus VX2 carcinoma

**DOI:** 10.3892/ol.2015.2899

**Published:** 2015-01-27

**Authors:** NA SHEN, XIUYIN XU, YAN SHA, HAITAO WU

**Affiliations:** 1 Department of Otolaryngology, Zhongshan Hospital, Fudan University, Shanghai 200032, P.R. China; 2 Department of Otolaryngology, Eye, Ear, Nose and Throat Hospital, Fudan University, Shanghai 200031, P.R. China; 3 Department of Radiology, Eye, Ear, Nose and Throat Hospital, Fudan University, Shanghai 200031, P.R. China

**Keywords:** VX2 carcinoma, neck lymph node metastasis, indirect computed tomography lymphography

## Abstract

Indirect computed tomography lymphography (CT-LG) could be used to determine the regional spread of cancer and assess lymphatic function by the interstitial delivery of diagnostic agents. Few studies have been reported on its use in pyriform sinus carcinoma. The aim of the present study was to establish the rabbit VX2 tumor as a model for pyriform sinus carcinoma and to observe its neck lymph node metastasis by indirect CT-LG. VX2 tumor tissue suspension was transplanted into the pyriform sinus submucosa of 15 rabbits under direct laryngoscope. Rabbits were randomly placed into one of three groups, each comprised of five rabbits. Observation of the tumor growth and neck lymph node metastases were taken on days 14 (group 1), 21 (group 2) and 28 (group 3) following transplantation using the method of indirect CT-LG. VX2 tumors were transplanted successfully in all rabbits. Deep cervical lymph nodes were enhanced clearly in indirect CT-LG. The contrast agent filling defected appeared on the metastasis nodes while the lymph node without metastasis was smooth. The metastasis rates of deep cervical lymph nodes were 100% in all three groups on CT-LG. The CT attenuation value of CT-LG reached peak values of 400 and 600 Hu at 1 and 3 min after the injection, which then decreased gradually. In this study, CT-LG could demonstrate the internal architecture of lymph nodes and their lymphatic vessels, and therefore may have the advantages of radiological methods such as B ultrasound, CT, magnetic resonance imaging and positron emission tomography.

## Introduction

Pyriform sinus carcinoma is one type of hypopharyngeal carcinoma. Early diagnosis of the disease is difficult, and lymph node metastasis can be observed in a number of patients (1). Therefore, lymph node status is one of the most important prognostic factors in pyriform sinus carcinoma (1). To date, neck lymph node dissection has been the standard procedure for lymph node metastasis (2). Although surgical dissection is an efficient therapeutic tool, it is accompanied by complications, including limitation of shoulder movement and lymph edema (2). In addition, some patients with pyriform sinus carcinoma have histopathologically negative neck lymph nodes. Thus, early diagnosis of the neck lymphatic metastasis in hypopharyngeal carcinomas has predominant value (3,4). During the past several years, this field gained increasing focus. B ultrasound, computed tomography (CT), magnetic resonance imaging and positron emission tomography remain the main methods. All the above methods demonstrate size rather than architecture, but it is occasionally not possible to discriminate whether lymphatic metastasis would happen when the lymph node is a normal size. The sentinel lymph node mapping is another method of early diagnosis (5). The most commonly used are the local injection of dye, lymphoscintigraphy imaging (5) and lymph node biopsy (6), but there are shortcomings. Thus, a new imaging technique that allows accurate preoperative assessment of the neck lymph nodes would be important. However, lymphography (LG) has the unique capability of demonstrating internal architectural derangements within normal-sized lymph nodes (7). The thin-walled and fenestrated lymphatic microvessel is easily penetrated by particulate and macromolecular agents following injection into the extracellular space. Once inside the vessel, materials that are transported with the lymph either specifically target certain nodal elements (e.g. neoplastic cells) or become cleared by macrophages located in the lymph nodes. According to this theory, interstitial delivery of diagnostic agents has been of benefit in determining the regional spread of cancer and assessing lymphatic function either by indirect CT-LG or indirect magnetic resonance imaging LG (8). This study focuses on indirect CT-LG used to identify lymph node metastasis in rabbit pyriform sinus VX2 carcinoma.

## Materials and methods

### Preparation of VX2 tumor mass suspension

The tumor cells (The First People’s Hospital of Shanghai Jiao Tong University, Shanghai, China) have been continuously passed intramuscularly in rabbits. Rabbits were anesthetized with 60 mg/kg ketamine (Jiangsu HengRui Medicine Co., Ltd., Nanjing, China) and 15 mg/kg pentobarbital sodium (Jiangsu HengRui Medicine Co., Ltd.) and the tumor cells were then implanted into the thigh muscle. Subsequent to the tumor growing to a palpable size, the rabbit was sacrificed using an intravenous overdose of pentobarbital (Jiangsu HengRui Medicine Co., Ltd.). The tumor was then excised. Muscle and necrotic tissue were removed. The tumor was placed in a petri dish with saline (Baxter Healthcare (Shanghai) Co., Ltd., Shanghai, China). Four 1-cm^3^ pieces were selected and cut with eye scissors into pieces ≤1 mm^3^. These pieces were then put into another dish with 40 ml balanced saline and suspended at 100 pieces/ml.

### Animal model

Fifteen New Zealand white rabbits, weighing 2.0–3.0 kg, were provided and bred by the Laboratory Animal Center of the Eye, Ear, Nose and Throat Hospital (Shanghai, China) under routine conditions according to the institute’s ethical and environmental guidelines. The rabbits were randomly divided into three groups, each containing five rabbits. Tumor implantations were performed under general anesthesia. The rabbits were premedicated with 60 mg/kg of 5% ketamine injected intramuscularly. After 5 min, a ‘butterfly’ needle (Shanghai Medical Instruments Co., Ltd., Shanghai, China) was inserted into the marginal vein of the ear, and 15 mg/kg pentobarbital sodium was injected slowly. Animals breathed without the aid of a respirator. Anesthesia was maintained ≤30 min with good analgesia and muscle relaxation.

Anesthetized rabbits were placed in a supine position on a specifically designed operation table (Eye, Ear, Nose and Throat Hospital) which allowed for the extension of the neck. A pediatric direct laryngoscope (Shanghai Medical Instruments Co., Ltd.) was used to expose the pyriform sinus. The VX2 mass suspension was drawn up into a 1-ml syringe (Jiangsu Zhengkang Medical Apparatus Co., Ltd., Changzhou, China) with a retropharyngeal puncture needle (1.2 mm diameter; Shanghai Medical Instruments Co., Ltd.) and 0.5 ml was injected into the submucosa of the lateral wall of the pyriform sinus. Injection into the site was confirmed by the visualization of a small mucosal bleb. The rabbits underwent a CT scan on days 14 (group 1), 21 (group 2), and 28 (group 3).

### CT

A CT scan was performed using a multi-detector raw CT scanner (SOMATOM Sensation 10, Siemens Healthcare, Shanghai, China). A CT scan was administered to each group. Each anesthetized animal was placed in the supine position on the CT table and tightly fixed with cotton tape, extending their neck. The CT scanning was operated at 120 kV, 150 mA, a 7–12 cm field of view, 512–512 matrix, with a detector of a 0.75 mm -10 rows, section spacing of 3 mm and table speed of 5.6 mm/r. The number of sections was individually adapted to ensure coverage of the anterior neck region.

Plain scan and venous enhancement with Omnipaque™ (GE Healthcare Inc., Princeton, NJ, USA)were conducted prior to indirect LG. Subsequently, 0.5 ml of Omnipaque™ was injected into the submucosa of the pyriform sinus bilaterally under the direct laryngoscope. The contiguous 3 mm-thick transaxial CT images from the submandibular regions to the supra-sternum regions were obtained immediately, in addition to successively at 1, 3, 5, 10 and 15 min after injection.

### Image analysis

One surgeon and one experienced radiologist analyzed the image data. The images were randomly viewed using a picture archiving and communication system workstation (PCVDMAPP MFC Application; Siemens AG Medical Solutions, Erlangen, Germany). The two doctors were blind to each other’s radiological evaluation results and to the histopathologic results. In cases of discrepancies between the two authors, a consensus was reached through discussion.

### Histological evaluation

Rabbits were scarified on days 14 (group 1), 21 (group 2) and 28 (group 3) after CT scanning. Prior to scarification, methylene blue (Southwest Synthetic Pharmaceutical Co., Ltd., Chongqing, China) mixed with Omnipaque™ was injected into the submucosa of the pyriform sinus bilaterally under the direct laryngoscope. Subsequently, a complete cervical lymph node dissection was performed and the pyriform sinus was observed and excised. The location and size of the resected lymph nodes were recorded for all rabbits. The blue-stained nodes were placed into the specimen bottle and subjected to a CT scan. The resected lymph nodes and pyriform sinus tumor were then fixed in 10% formalin (Hubei Xinfei Chemical Co.Ltd, Wuhan, Hubei, China)in saline for ≥24 h. Sections were stained with hematoxylin and eosin (Beyotime Biotechnology (Shanghai) Co., Ltd, Shanghai, China).

### Statistical analysis

SPSS 11.5 software (SPSS, Inc., Chicago, IL, USA) was used for data analysis. The average diameter of lymph nodes between the CT and histological evaluation was compared by the way of paired-sample t-test. A significance level of α=0.05 was used.

## Results

### CT study

All the rabbits taken from the three groups were scanned by CT. Eight tumors were found in the left hemisphere while seven tumors were found in the right. In group 1, soft tissue images could be observed in the implanted side, and the pyriform sinus disappeared. However, paraglottic space invasion could also be observed. In group 2, images of thyroid cartilage destruction and outside invasion of tumors could be observed. In group 3, the tumors could be observed adhering to adjacent nodes.

**Figure 1:**
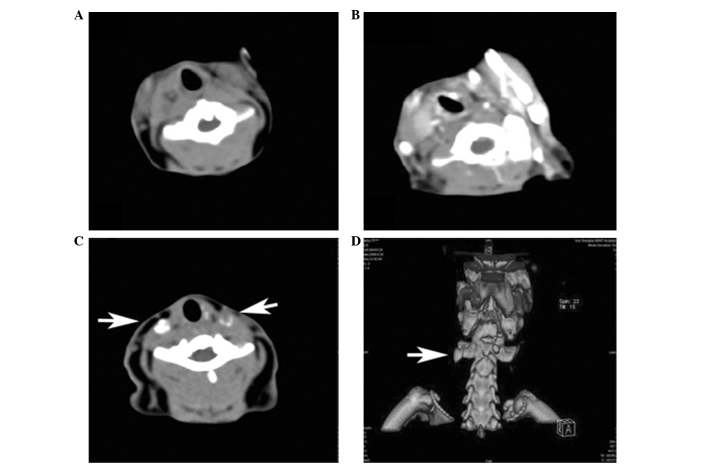
Imaging findings of cervical lymph nodes. (A) On plain CT images, cervical lymph nodes were not clearly identified. (B) Cervical lymph nodes were also not clearly identified in the venous enhancement. (C) By CT lymphography, the contrast agent filling defected appeared on the metastasis nodes. (arrow) (D) The oval-shaped lymph nodes (arrow) with greater diameters than those of the lymphatic vessels were easily identified on three dimensional images. CT, computed tomography.

**Figure 2:**
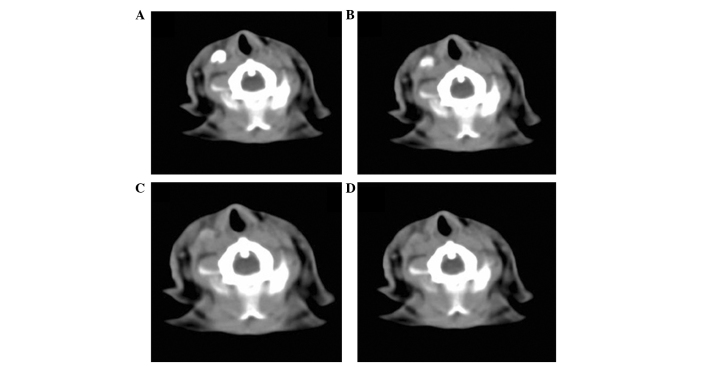
CT attenuation changed with the time following injection. Images at (A) 3 min (highest CT attenuation), (B) 5 min, (C) 10 min and (D) 15 min (<50 HU). CT, computed tomography.

On plain CT images, cervical lymph nodes were not clearly identified in all the rabbits (Fig. 1A). This also occurred in the venous enhancement with a slightly high density (Fig. 1B). However, following the injection of Omnipaque™ into the submucosa of the pyriform sinus bilaterally, the deep cervical lymph nodes were rapidly enhanced in each rabbit. However, the submandibular cervical lymph nodes remained unrevealed. Ipsilateral deep lymph node metastasis was observed in 100% of animals in all the groups, and contralateral metastasis occurred at rates of 80% on day 14, 60% on day 21, and 100% on day 28. There was one oval-shaped enhanced lymph node which was located lateral to the larynx and cricoid cartilage, below the sterno-thyroid muscle. The contrast agent filling defected appeared on the metastasis nodes while the lymph node without metastasis was smooth (Fig. 1C). Time points of 1 and 3 mins after injection exhibited the highest CT attenuation, ranging at 400–600 HU. The CT attenuation generally decreased from 5 min with a range of 200–400 HU, to 50–100 HU at 10 min and <50 HU at 15 min (Fig. 2A–D). Three-dimensional (3D) CT images were reconstructed from the CT-LG images at each time point. The oval-shaped lymph nodes with greater diameters than those of the lymphatic vessels were easily identified (Fig. 1D). Thus it effectively displayed the vessels from the submucosa of the pyriform sinus directed to each node located in the para-laryngeal areas. Unsmoothed surfaces in the metastasis nodes were easily observed.

### Gross appearance and histology

**Figure 3:**
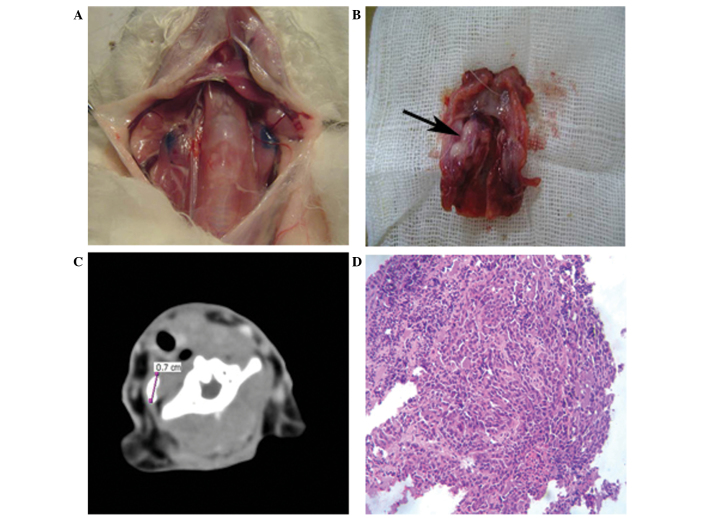
Pathological and imaging findings. (A) Blue-stained nodes, one for each side. (B) Tumors were visible in the pyriform sinus at the primary site (arrow). (C) The diameter of the lymph node was measured in the computed tomography image. (D) Lymph node metastasis of poorly differentiated squamous cell carcinoma. (hematoxylin and eosin; magnification, ×200).

Deep cervical lymph nodes were located laterally, with 1–2 oval lymph nodes per side, into the larynx and cricoid cartilage below the sterno-thyroid muscle. However, the blue-stain nodes were only one for each side (Fig. 3A). Following a subsequent CT scan, the nodes were observed to be enhanced. The other two groups of nodes, including the para-tracheal and sub-mandibular lymph nodes, could not be observed with the blue-stain. The occurrence of the ipsilaterally located deep lymph node metastases was observed in 100% of the animals in all the groups. Contralateral metastasis rates were 80% on day 14, 60% on day 21 and 100% on day 28 (Table I). It was in accordance with the CT image. The median diameter of the nodes on the CT images was 0.533±0.056 cm, while 0.541±0.066 cm by the histological method. No significant differences were found between histology and CT scanning (Fig. 3C).

Tumors were visible in the pyriform sinus at the primary site (Fig. 3B). Eight tumors were placed on the left side, while seven were inoculated at the right side. Although all tumors were implanted submucosally, five rabbits in group 1 still showed surface ulceration and paraglottic space invasion. In group 2, we found thyroid cartilage destruction and invasion of the tumor outside of the sinus. On day 28, the tumors were found to adhere to adjacent nodes. However, the medial wall and contralateral pyriform sinus were not invaded. Since it was difficult to discriminate the tumor from its surrounding tissues, the present study did not statistically analyze the diameters of the tumors. Microscopically, the tumors were poorly differentiated squamous cell carcinoma and invaded the pyriform sinus, with some showing multi-focal necrosis (Fig. 3D).

## Discussion

Three types of CT scan method were used in this study, which were scanning without contrast agent, venous contrast and CT-LG. On plain CT images, cervical lymph nodes were not clearly identified in all rabbits. This also occurred in the venous enhancement, even for the lymph nodes >1 cm. This may be due to the contrast agent through the blood circulation, with components of the metabolic loss. By diffusion into the lymphatic system, its concentration is lower in the lymphatic system than in the blood. However, deep cervical lymph nodes were clearly enhanced with CT-LG. This phenomenon reflects the advantages of indirect LG. The contrast agent filling defected could be observed on metastasis nodes. The vessels directing from tumors to lymph nodes were developed on 3D images. This can provide information on the internal structure and drainage of lymph nodes.

In addition, indirect CT-LG can be used to identify sentinel lymph nodes. Torchia *et al* (9) reported the detection of sentinel lymph nodes of pig tongue carcinomas. In the present study, a rabbit animal model was used. Since the distribution of rabbit cervical lymph nodes is similar to that of humans (10), this is a good model for lymph node metastasis research. In the rabbit model of auricular and tongue VX2 carcinoma, the sentinel lymph nodes are same as the parotid lymph nodes (11,12). Our previous study indicated that pyriform sinus carcinoma metastasizes primarily in the deep cervical lymph nodes (13). By analysis of the CT-LG image, it was revealed that superficial cervical lymph nodes still could not be revealed. This also occurred in the histological evaluation with methylene blue injection. The deep cervical lymph nodes were the first group of lymph nodes to develop metastases. According to this, deep cervical lymph nodes could be concluded as the sentinel lymph nodes of pyriform sinus carcinoma. This was in accordance with our former study, and also with the viewpoint of the study by Dunne *et al* (10).

Furthermore, the present study revealed that the numbers of the lymph nodes identified on the CT scan were quite different from those identified by histology. This may be due to the contrast agent concentration and infusion rate in the second leg of the lymph nodes not achieving the imaging requirements. This phenomenon can be attributed to the nature of the contrast agent. The present study used the water soluble non-ionic contrast agent Omnipaque™, which exhibited a shorter reaction time. The peak appeared immediately following injection (1 and 3 min), and at 5 min began to disappear. This finding is consistent with the study by Suga *et al* (14). Contrast agent combined with lymphatic system carrier could be used to extend developing time due to its larger molecular weight (15). Therefore, more focus should be given to studies on developing contrast agents, as this may result in improved use of indirect LG. This study presents the preliminary experience of indirect CT-LG in rabbit pyriform sinus carcinoma, which may be valuable to further clinical studies.

## Figures and Tables

**Table I:** Number of lymph nodes at different times following computed tomography (CT) lymphography.

		Deep cervical lymph nodes		Filling defected		Smoothed	
				
Group	n	Ipsilateral	Contralateral	Ipsilateral	Contralateral	Ipsilateral	Contralateral
1	5	5	5	5	4	0	1
2	5	5	5	5	3	0	2
3	5	5	5	5	5	0	0

Groups: 1, CT scan on day 14; 2, CT scan on day 21; 3, CT scan on day 28.
